# Methyl-Trimethoxy-Siloxane-Modified Mg-Al-Layered Hydroxide Filler for Thermal-Insulation Coatings

**DOI:** 10.3390/ma16124464

**Published:** 2023-06-19

**Authors:** Yanhua Zhao, Guanhua Shen, Yongli Wang, Xiangying Hao, Huining Li

**Affiliations:** 1 Guangdong Provincial Key Laboratory of Environmental Health and Land Resource, College of Environmental and Chemical Engineering, Zhaoqing University, Zhaoqing 526061, China; 2017010008@zqu.edu.cn (Y.Z.); shenguanhua@zqu.edu.cn (G.S.); 2021010065@zqu.edu.cn (Y.W.); 2 Zhaoqing Rivers High-Tech Materials Co., Ltd., Zhaoqing 526061, China; 13902398773@163.com

**Keywords:** Mg-Al-layered double hydroxide, thermal insulation, water-based coatings, building energy efficiency

## Abstract

The development of high-performance insulation materials that facilitate the reduction in building energy consumption is of paramount significance. In this study, magnesium–aluminum-layered hydroxide (LDH) was prepared by the classical hydrothermal reaction. By implementing methyl trimethoxy siloxane (MTS), two different MTS-functionalized LDHs were prepared via a one-step in situ hydrothermal synthesis method and a two-step method. Furthermore, using techniques, such as X-ray diffraction, infrared spectroscopy, particle size analysis, and scanning electron microscopy, we evaluated and analyzed the composition, structure, and morphology of the various LDH samples. These LDHs were then employed as inorganic fillers in waterborne coatings, and their thermal-insulation capabilities were tested and compared. It was found that MTS-modified LDH via a one-step in situ hydrothermal synthesis method (M-LDH-2) exhibited the best thermal insulating properties by displaying a thermal-insulation-temperature difference (ΔT) of 25 °C compared with the blank panel. In contrast, the panels coated with unmodified LDH and the MTS-modified LDH via the two-step method exhibited thermal-insulation-temperature difference values of 13.5 °C and 9.5 °C, respectively. Our investigation involved a comprehensive characterization of LDH materials and coating films, unveiling the underlying mechanism of thermal insulation and establishing the correlation between LDH structure and the corresponding insulation performance of the coating. Our findings reveal that the particle size and distribution of LDHs are critical factors in dictating their thermal-insulation capabilities in the coatings. Specifically, we observed that the MTS-modified LDH, prepared via a one-step in situ hydrothermal approach, possessed a larger particle size and wider particle size distribution, resulting in superior thermal-insulation effectiveness. In contrast, the MTS-modified LDH via the two-step method exhibited a smaller particle size and narrow particle size distribution, causing a moderate thermal-insulation effect. This study has significant implications for opening up the potential for LDH-based thermal-insulation coatings. We believe the findings can promote the development of new products and help upgrade industries, while contributing to local economic growth.

## 1. Introduction

With escalating energy scarcity across the country, the conservation of energy and the reduction in consumption have become a collective concern for the whole of society. In the field of construction, the utilization of insulating materials to curtail the energy loss in buildings has emerged as a pivotal endeavor [1,2,3,4,5]. The retrofitting of buildings to optimize energy usage constitutes an indispensable aspect of sustainable development. While adding thermal insulating materials to a building envelope is one of the most conventional and familiar approaches, prevailing energy-saving retrofitting techniques do not account for the ecological impact of such materials. Traditional inorganic materials for thermal insulation, such as asbestos, mineral wool, and expanded perlite, etc., exhibit charming thermal insulating properties but are detrimental to the environment [6]. Their alternative organic analogs, e.g., polymer hair foam and foam asbestos insulation materials, etc., unfortunately possess considerable volume and thus entail significant transport and construction expenditures. Consequently, numerous novel insulation materials have been developed to enhance the energy-conservation performance of the existing and new constructions. These materials provide enhanced thermal-insulation properties [7,8,9], enhance economic viability, and promote carbon neutrality. It is clear that the development of high-performance insulation materials that facilitate the reduction in building energy consumption is of paramount significance [10].

The wide utilization of winter heating and summer cooling equipment in global high-energy-consuming construction exacerbates energy wastage. Nonetheless, the application of water-based thermal-insulation coating sprayed onto surfaces can effectively deliver a cooling and heat-insulating impact [11]. Concurrently, the water-based coating is a coating that entails water as the dispersion medium and is eco-friendly and non-toxic. This approach can accomplish the objective of energy conservation and emission reduction. To date, thermal-insulation coatings can be classified into four categories based on their heat-insulation mechanisms, namely the barrier type, the reflective type, the radiation type, and the composite type. Among them, reflective coatings can effectively obstruct heat conduction, and their reflective properties depend on the reflectivity of the coating surface to a great extent [12,13,14]. Numerous factors have a significant impact on the reflectivity of the coating, e.g., the ratio between the refractive coefficient of the filler and the coating resin matrix, the coating film thickness, and the particle size and particle size distribution of the filler, etc. [15,16].

As an important inorganic filler, hydrotalcite can enhance the thermal-insulation effect of the coatings effectively. Hydrotalcite (LDH) is an inorganic material with a layered bimetallic hydroxide structure, which is utilized in various applications [17,18,19,20,21,22,23]. Its small specific gravity and low thermal conductivity make it suitable for use in barrier-type thermal-insulation coatings. Additionally, LDH has shown excellent thermal insulation, UV reflection, infrared absorption, and infrared scattering functions. Moreover, after calcination at a specific temperature, LDH can further enhance thermal insulation and become more suitable as reflective thermal-insulation materials. Therefore, LDH has great potential for extensive application in water-based thermal-insulation materials. As a mineral substance, hydrotalcites are easily affordable and have many manufacturing possibilities. The availability of sufficient natural deposits also means that hydrotalcites have great potential for application in the construction industry, as a building material or as a filler in coatings. Sun Yao et al. studied the impact of hydrotalcites on the thermal-insulation performance of interior wall coatings and found that hydrotalcites could cause a temperature change in the system by 5 °C via infrared blocking. However, there are limited reports on the use of hydrotalcites in the preparation of thermal-insulation coatings.

In this study, magnesium–aluminum-layered hydroxide (LDH) was prepared through the classical hydrothermal method, and two different LDHs were also produced by modification with methyl trimethoxy siloxane (MTS), using either the one-step in situ hydrothermal synthesis method or the two-step method. Chemical modification is demonstrated not only by experiments, but also by theoretical methods, which play a crucial role in regulating the properties/structure of materials of similar complexity [24,25,26,27]. The MTS-modified LDHs were then utilized as fillers in waterborne coatings, and their thermal-insulation and heat-retention performances were evaluated and compared. By conducting a series of characterizations of LDH and coatings, the thermal-insulation and heat-preservation mechanisms were explored. In addition, the correlations between the LDH particle size (as well as the particle size distribution) and the thermal insulation and heat-preservation performance of the coatings thereof are studied [28]. This study is significant in establishing a new type of thermal-insulation coating with LDH-based functional fillers, which can be leveraged towards conserving energy consumption.

## 2. Experimental

### 2.1. Materials

The raw materials, including sodium carbonate, sodium hydroxide, magnesium nitrate hexahydrate, and aluminum nitrate nonahydrate, were all purchased from Xi long Chemical Co. Ltd. (Guangdong, China). The film-forming additive was purchased from American Angus Corporation (Buffalo Grove, IL, USA). The acrylic resin was acquired from Zhao Qing Rivers High-Tech Materials Co. Ltd. (Zhaoqing, China). Methyl trimethoxy siloxane (MTS) was commercially obtained from Jinan Xing Fei Long Chemical Co., Ltd. (Jinan, China).

### 2.2. Preparation of Mg-Al Hydrotalcite (LDH)

Prior to the synthesis of LDH, a solution consisting of 19.2 g of Mg(NO_3_)_2_·6H_2_O and 10.6 g of Al(NO_3_)_3_·9H_2_O was dissolved in 160 mL of water (referred to as solution A). Separately, a solution containing approximately 8 g of NaOH and 8.6 g of Na_2_CO_3_ dissolved in 60 mL of water was also prepared (referred to as solution B). Solution B was added into solution A dropwise under vigorous stirring, while the pH value was maintained at approximately 10. The resulting mixture was then transferred into a hydrothermal autoclave reactor and stirred continuously at 120 °C for another 6 h. After the reaction was complete, the resultant precipitate was then filtered, washed with distilled water, and dried at 80 °C for 12 h. The white solid product yielded is denoted as LDH.

### 2.3. Preparation of MTS-Modified LDH (M-LDH-1)

M-LDH-1 was prepared via a tow-step method as follows: to a suspension consisting of 50 g of LDH material prepared as described above and 200 mL of water, a specific amount of MTS (1.5 g, 3 wt% to LDH) was added under vigorous stirring. The suspension was stirred continuously in a water bath at 80 °C for 1 h. After the reaction was complete, the precipitate was filtered, washed with distilled water, and dried at 80 °C for 12 h. The obtained white solid product is denoted as M-LDH-1.

### 2.4. Preparation of MTS-Modified LDH (M-LDH-2)

The MTS-modified LDH one-step in situ hydrothermal synthesis method [29] was prepared as follows: a solution consisting of 19.2 g of Mg(NO_3_)_2_·6H_2_O and 10.6 g of Al(NO_3_)_3_·9H_2_O was dissolved in 160 mL of water (referred to as solution C). Another solution containing approximately 8 g of NaOH and 8.6 g of Na_2_CO_3_ dissolved in 60 mL of water was also prepared (referred to as solution D). In addition, an MTS aqueous solution with a pH value of 4–5 was also prepared by dissolving a pre-determined amount of MTS (0.25 g, 3 wt% to LDH) in 10 mL of water (referred to as solution E). Solution D was added into solutions C and E dropwise under vigorous stirring at an ambient temperature. The mixture was then transferred into a hydrothermal autoclave reactor and stirred using the same procedure as in the LDH synthesis described above. The formatted precipitate was then filtered, washed with distilled water, and dried at 80 °C for 12 h. The obtained white solid product is denoted as M-LDH-2.

### 2.5. Preparation of Thermal Insulating Coating Samples

The preparation of the thermal insulating coating was based on the formulation depicted in Table 1. To prepare the coating samples, pre-weighed acrylic resin and film-forming additive were mixed according to the formulation shown in Table 1. The mixture was then blended for around 6 min at a low stirring speed to achieve even dispersion. Subsequently, filler and water were introduced into the mixture gradually. The mixture was stirred vigorously for an additional 20–35 min under a high stirring speed to disperse the components evenly. By incorporating a pre-determined amount of curing agent, the coating samples were then finalized.

### 2.6. Preparation of Waterborne Thermal Insulating Films

The preparation of the coating film was based on the standard coating preparation method of GB/T1765-1979. The thermal insulating films were prepared by dropping the prepared coating samples described above onto the asbestos sheet. The films were applied evenly on the surface of the asbestos sheet using a 150 μm wire rod. The film samples were dried, and the thermal-insulation performance was evaluated.

### 2.7. Characterizations

The powder XRD patterns of the LDHs were recorded on a Bruker D8 Advance diffractometer with Cu Kα radiation (k = 1.5406°), operating at 40 kV and 40 mA. The samples were measured in the 2θ range of 5–70° with a scan rate of 3°/min. Fourier-transform infrared (FTIR) spectra were acquired using infrared spectroscopy (FTIRracer-100, Shimazu, Kyoto, Japan) in the spectral range of 400–4000 cm^−1^ with a resolution of 4 cm^−1^, using the KBr pellet technique. The morphology observations were conducted on a scanning electron microscope (SUPRA55, Zeiss, Jena, Germany), operating at 30 kV and 10.0 μA. UV–Vis diffuse reflectance spectra were recorded on a spectrophotometer with an integrating sphere (UV-2600, Shimadzu, Kyoto, Japan). The reference standard reflectance material used for the measurement was BaSO_4_. The particle size distributions were measured using a laser granulometer (LA-960S2, HORIBA. Ltd., Kyoto, Japan).

### 2.8. Thermal-Insulation Performance Evaluation

**Figure 1:**
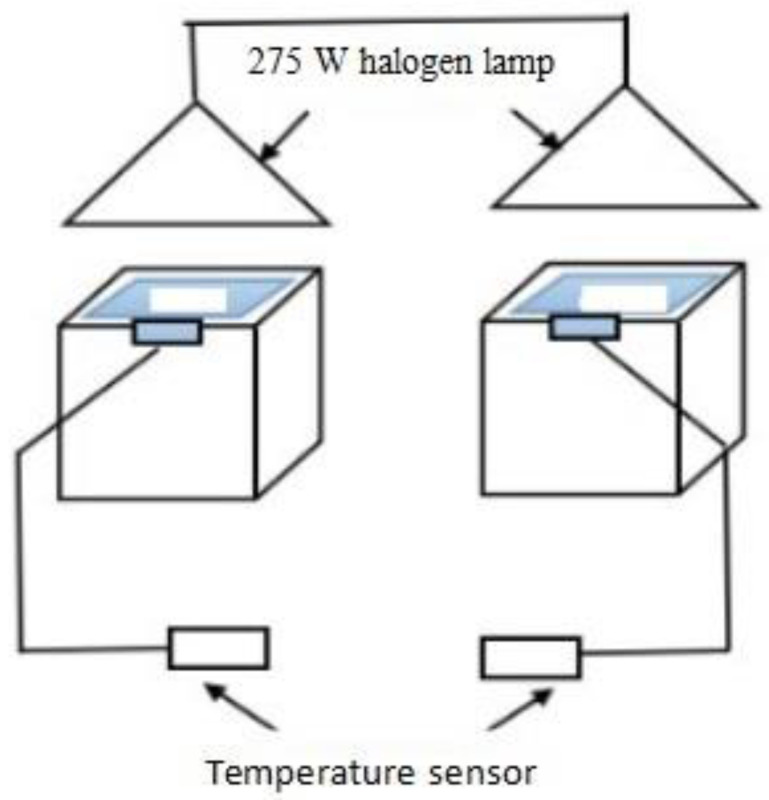
Schematic illustration of the custom-built thermal-insulation testing device.

Thermal-insulation performance evaluation of the film samples was conducted on a custom-built thermal-insulation testing device, as depicted in Figure 1. The thermal-insulation test for the coating samples utilized a 275 W halogen lamp as the light source and a flat asbestos fiber cement panel as the test panel. The temperatures of the back side of each asbestos slab were recorded by temperature sensors every 5 min over a test span of 45 min. The thermal-insulation performance of the coating samples was ascertained by measuring the temperature of the asbestos sheet without any coating (T_blank_) relative to the temperature of the asbestos sheet coated with the coating sample (T_film_). The thermal-insulation performance was then obtained by calculating the thermal-insulation-temperature difference, ΔT = T_blank_ − T_film_.

## 3. Results and Discussion

### 3.1. Structure Characterizations

**Figure 2:**
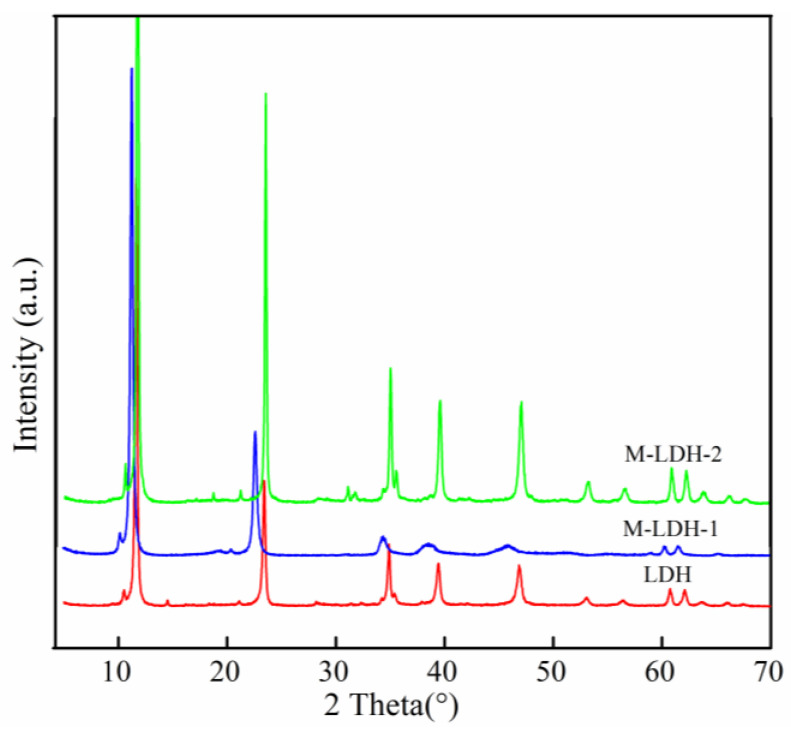
XRD patterns of LDH (down), M-LDH-1 (middle), and M-LDH-2 (up).

The X-ray diffraction patterns of the pristine LDH and the MTS-modified LDHs are illustrated in Figure 2. As shown in Figure 2 (down), the XRD pattern of the original LDH sample exhibits a well-ordered, typical layered structure with a basal spacing (d_003_) of 7.9 Å, corresponding to CO_3_^2−^ (Mg-Al-CO_3_^2−^-LDH). This value aligns well with the standard ICDD reference pattern 00-022-0700 (hydrotalcite, syn-Mg_6_A_l2_(OH)_16_CO_3_·3H_2_O). MTS-modified LDHs obtained by both modification methods (Figure 2 (middle) and Figure 2 (up)) also present narrow, sharp patterns with high diffraction intensity within the 2θ range of 7–37° (d_003_, d_006_, and d_009_), as well as similar d_00l_ values to those of the original LDH. In addition, two sharp, symmetrical, and lower reflections within the 2θ range of 60 to 63° (d_110_ and d_113_) emerge, indicating the high crystallinity of the resulting LDH materials. The consistency in the d-spacings illustrates that both modification methods have little impact on the chemical structures of the LDH. In our study, we also observe shifts in the peaks at d003, d006, and d009 of the modified LDH compared to the pristine LDH, particularly in M-LDH-1. These shifts are primarily attributable to the insertion of MTS into the basal spacing of LDH. Notably, M-LDH-2 exhibits a higher peak intensity than both pristine LDH and M-LDH-1, suggesting the greatest degree of crystallinity. Our findings suggest that the one-step in situ method does not significantly alter the structure of the LDH due to surface reactions between MTS and LDH, without the insertion of MTS into the interlayer of LDH. Additionally, we observe that MTS on the exterior of LDH can decrease viscosity, leading to enhanced crystallinity of LDH. This has important implications for the practical application of modified LDH in nanocomposite materials.

**Figure 3:**
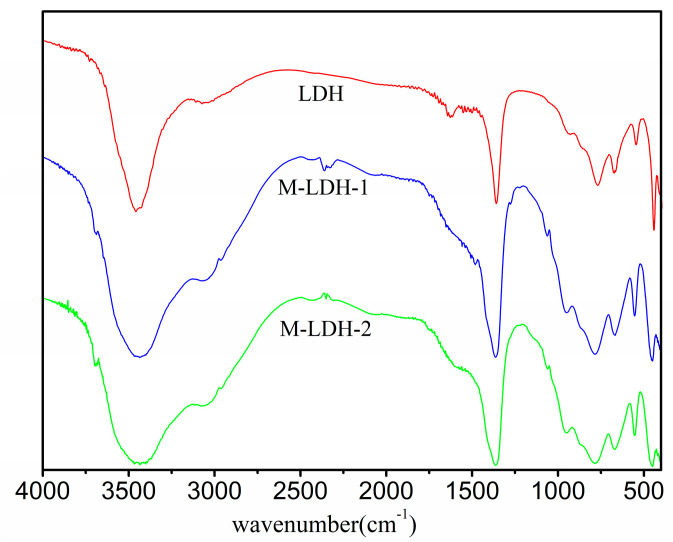
FTIR spectra of LDH, M-LDH-1 and M-LDH-2.

The alterations in bonding between the original layered double hydroxide (LDH) and the MTS-modified LDH samples were verified by Fourier-transform infrared (FTIR) spectroscopy, as depicted in Figure 3. For the original LDH, the hydroxyl group (-OH)-related bands are located at 3466 cm^−1^ (water) and 3073 cm^−1^ (the bridge bond between H_2_O and anions). The bending mode associated with water is observed at approximately 1636 cm^−1^. The sharp and intense band present at around 1350 cm^−1^ arises due to the asymmetric stretching mode of CO_3_^2−^, while the band at 827 cm^−1^ is assigned to v_2_ modes of the CO_3_^2−^ located within the interlayer spaces. The bands at 576 cm^−1^ are attributed to the M–O stretching vibration (M = Mg^2+^, Al^3+^) [29]. These bands of the two MTS-modified LDHs (M-LDH-1 and -2) are in agreement with that of the original LDH sample, indicating that the LDH matrix structures stay intact by the two modification methods, as corroborated by the XRD results. Notable, a weak new band emerges at approximately 1060 cm^−1^ for M-LDH-1 and -2, which corresponds to the Si-O-Si vibration of the MTS. The FTIR spectra clearly confirm the successful modification of LDH by MTS.

**Figure 4:**
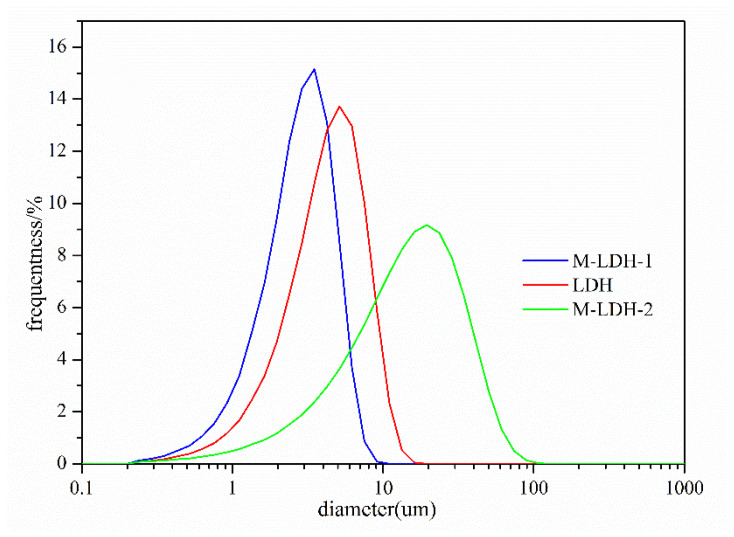
Particle size distribution curves of pristine LDH (red), M-LDH-1 (blue), and M-LDH-2 (green).

It is generally recognized that the particle size and distributions of the inorganic fillers have a direct impact on the thermal-insulation properties of the final product. The particle sizes of three LDHs were measured, and the results are presented in Figure 4. The original LDH exhibits a relative broad particle size distribution, with an average particle size of around 9 μm. In a similar fashion, the M-LDH-1 sample also displays a relative broad particle size distribution, and its average particle size is approximately 5 μm. In sharp contrast, the M-LDH-2 sample displays a much wider particle sizes distribution, with an average size of around 25 μm. Clearly, the in situ modification method leads to the largest particle size with a wide particle size distribution, while the two-step modification method resulted in a much smaller and narrow particle size distribution. This is most likely due to the fact that MTS reacts with the facial hydroxyl functionalities and binds the LDH crystals by forming a polysiloxane network. This leads to the formation of an intermolecular Si-O-Si linkage matrix between the two or more adjacent silylated LDHs at the neighborhood [30,31]. In sharp contrast, if the reaction occurs using the two-step modification method, only the silylated reaction with facial -OH of LDH particles would take place. As a result, no aggregation of LDH takes place, and the clusters of LDH may be separated by the mechanical stirring and cutting.

**Figure 5:**
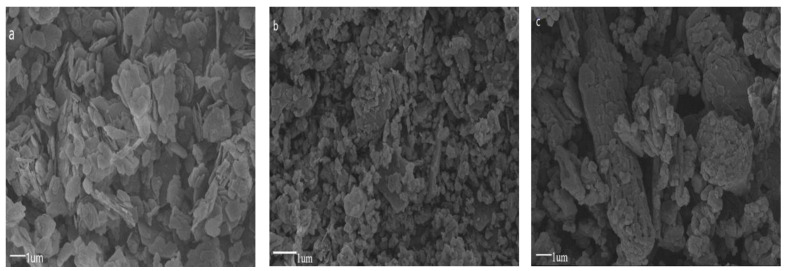
SEM images of the pristine LDH (**a**), MTS-modified samples of M-LDH-1, (**b**) and M-LDH-2 (**c**).

To observe the morphology of LDH and MTS-modified LDHs samples at a microscale, scanning electron microscope (SEM) observation was performed, and the SEM images of LDH, M-LDH-1, and M-LDH-2 are presented in Figure 5. As shown by the SEM image of the pristine LDH in Figure 5a, the unmodified LDH powder aggregates into small particles with a plate-like morphology of a hexagonal lamellar structure with rounded corners. The particle size is around 9 μm, which is consistent with the particle size measurement shown in Figure 4. Upon modification of the LDH samples using the two-step method (M-LDH-1), the smooth flakes are transformed into irregular, small, and separate particles, with a diameter of approximately 5 μm (Figure 5b). The diameter of M-LDH-1 also agrees with the particle size measurement shown in Figure 4. In contrast, the LDH sample modified using the in situ method (M-LDH-2) exhibits a various degree of agglomerations, with relatively regular and orderly layer combinations. In addition, the layers are also packed together closely, resulting in the formation of “rod-like” pillars, with a length of about 25 μm (Figure 5c). Once again, the particle size of M-LDH-2 observed in SEM observations confirmed the particle size measurement shown in Figure 4. Obviously, the crystals of the LDH sample modified using the in situ method tend to bond with each other. It is evident from the SEM images of the LDH and MTS-modified samples using the two-step method and the in situ method that the modification method has a significant impact on the morphology of LDH products.

**Figure 6:**
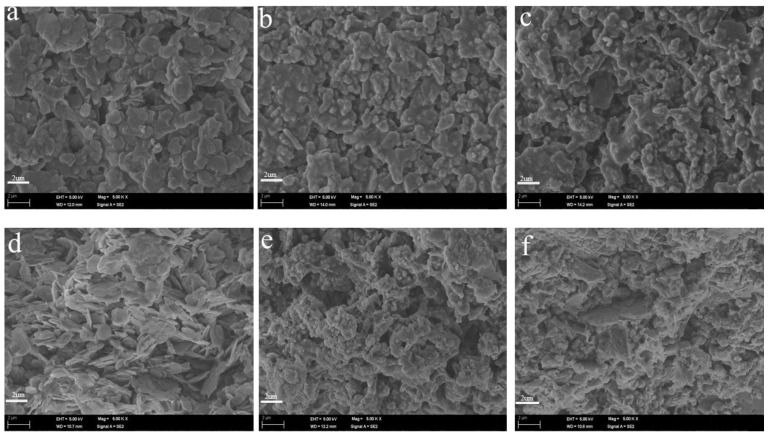
Facial and cross-sectional SEM images of coating films with LDHs. (**a**–**c**) The facial SEM images of the coating films with LDH (**a**), M-LDH-1 (**b**), and M-LDH-2 (**c**); (**d**–**f**) The cross-sectional SEM images of the coating films with LDH (**d**), M-LDH-1 (**e**), and M-LDH-2 (**f**).

The compatibility between the LDH particles and the coating resins is critical in forming high-performance thermal-insulation materials. To further explore the compatibility of LDH modified using the two different methods in the coating system and the microscale factors leading to thermal-insulation variations, the SEM observation of the coating films with different LDHs was conducted. The facial and cross-sectional SEM images of the coating films with different LDHs are depicted in Figure 6. As illustrated in Figure 6a,b, the pristine LDH particles in the coating system appear to have typical flake morphology, which is basically incompatible with the coating resin. Macroscopic phase separation was observed in the unmodified LDH. However, our investigations revealed that both MTS-modified LDH samples (M-LDH-1 and -2) synthesized via two distinct methods exhibit remarkable compatibility with the coating system, as exemplified by the complete wetting and wrapping of the LDH filler by the resin (Figure 6c–e). This observation highlights the beneficial effect of MTS modification on the compatibility of LDH with the coating matrix, facilitating the development of high-performance composites.

### 3.2. Thermal-Insulation Properties and NIR Reflectance

#### 3.2.1. Thermal-Insulation Properties

**Figure 7:**
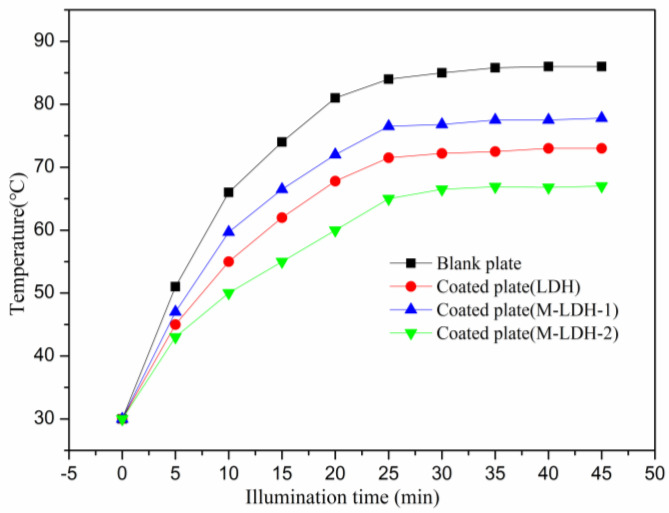
The T_eq_ variations of the plates with coatings with different LDHs are plotted as a function of time.

The thermal-insulation properties of the coating films with LDHs were characterized using the custom-made custom-built thermal-insulation testing device, as depicted in Figure 1. The tests were conducted starting from room temperature until equilibrium temperatures (T_eq_) were reached. Herein, the equilibrium temperature is defined as the temperature with minimal temperature fluctuations (<1 °C) observed over a 5 min period. All the tests were carried out under the same illumination conditions, starting at the same initial temperature of 30 °C [32]. As depicted in Figure 7, the variations in temperature are recorded at the back surface of the cement reference panel covered with thermal-insulation coatings, and an LDH-free coating is used as a control. Under iodine-tungsten light, the back surface temperature of the blank panel without thermal-insulation coatings exhibits a rapid increase in temperature and reached a T_eq_ value of 85.4 °C after approximately 35 min. In comparison with the blank plate, the cement reference panels coated with different types of LDH-containing thermal-insulation coatings demonstrates a noteworthy decrease in temperature on the back surface, indicating their good thermal-insulation performance. Among the LDH-containing coatings, the coating with M-LDH-2 shows the best thermal-insulation effect, with a much lower T_eq_ value of about 65 °C and a much slower temperature increasing rate. The panels with LDH and M-LDH-1 exhibit similar and minor improvements in terms of thermal-insulation properties, with T_eq_ values of 72 °C and 76 °C, respectively. These results clearly demonstrate the effectiveness of employing the MTS-modified LDH filler with respect to applications as thermal-insulation materials. Interestingly, the thermal-insulation performance test demonstrated that larger particle size and wider particle size distribution have a significant impact on the thermal-insulation performance of LDH-containing coatings [33]. The thermal conductivity of composite materials is predominantly influenced by the conductivity of the continuous and dispersed phases. In our study, we incorporated a modified LDH filler as the dispersed phase and utilized a cured polyacrylic resin matrix as the continuous phase. We envision that fillers with smaller particle sizes form a more continuous pathway through-out the material, resulting in a significant decrease in thermal conductivity and improved insulation properties. This finding highlights the importance of carefully selecting the filler particle size in the design of high-performance thermal-insulation coatings. In addition, the compatibility of the LDH filler in the coating matrix has no direct effect on the thermal-insulation performance as LDH and M-LDH-1 exhibit similar T_eq_ values.

#### 3.2.2. UV–NIR Reflectance

**Figure 8:**
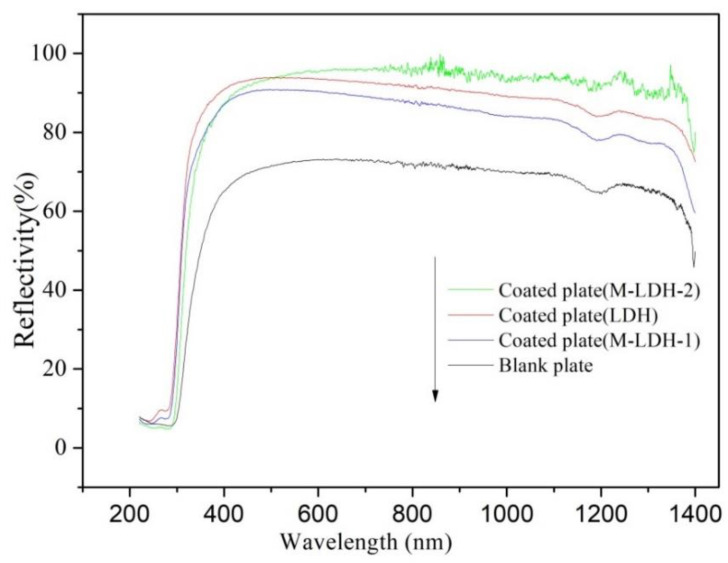
UV–NIR spectral reflections of the coatings containing different LDHs as a function of wavelength.

In order to investigate the thermal-insulation mechanism, UV–NIR diffuse reflection analysis of the coating films with different LDH fillers was conducted. The UV–NIR spectral reflections of all the samples are plotted as a function of wavelength, as depicted in Figure 8. It is evident that the UV–NIR spectral reflections of the coatings containing LDHs are all significantly higher in comparison with that of the blank plate without any thermal-insulation coatings. Notably, the UV–NIR spectral reflectance of the coating with M-LDH-2 is significantly higher than that of coatings with M-LDH-1 and LDH. This enhancement of M-LDH-2-containing coatings could be attributed to the increased particle size and particle size distributions, as well as the resultant densely packed layers due to the in situ modification reaction on the LDH. As a result, the addition of M-LDH-2 to the coating system results in a higher reflectivity of the coated plate. Furthermore, the UV–NIR spectral reflections of coatings with M-LDH-1 is less than that of the coating with LDH, which is possibly due to the modification of LDH by the two-step method, leading to a reduction in particle size. Consequently, the addition of M-LDH-1 to the coating system decreases more in the reflectivity of the coated plate compared with unmodified LDH. It can be inferred that the particle size of LDH plays a critical role in UV–NIR light-reflection efficiency, and the increase in UV–NIR reflectivity of the coating may be attributed to an increase in the particle size of the added LDH sample. To effectively reflect and block thermal and energy radiations, coatings should possess high solar reflectance. This is in line with prior research that highlights the importance of high solar reflectance in coatings for streamlining thermal-insulation efficiency [34,35,36].

## 4. Conclusions

In this study, LDHs were successfully synthesized via the classical hydrothermal method. Additionally, MTS-modified LDHs were successfully prepared using either the one-step in situ hydrothermal reaction or the two-step method. The successful synthesis and modification reaction are confirmed by the FTIR measurements. As disclosed by XRD, the crystal structures of the LDHs stay intact when performing the MTS modification reaction using the two different methods. The pristine LDH exhibits a relative broad particle size distribution, with an average particle size of around 9 μm. The M-LDH-1 sample also displays a relative broad particle size distribution, and its average particle size is approximately 5 μm. The M-LDH-2 sample displays a much wider particle size distribution, with an average size of around 25 μm. The SEM observation confirms the particle size detection, and M-LDH-2 exhibits a various degree of agglomerations, with relatively regular and orderly layer combinations, forming “rod-like” pillars. Both the M-LDH-1 and -2 sample exhibits enhanced compatibility with the coating resin matrix by MTS modification, while the pristine LDH shows less compatibility. When LDHs are applied as the inorganic fillers in thermal-insulation coatings, different LDHs exhibits varying thermal-insulation effects. The panel with thermal-insulation coating with M-LDH-2 displays the best thermal-insulation performance. T_eq_ exhibited by the panel coated with M-LDH-2-containing thermal-insulation coating is 65 °C, which is 25 °C lower than that of the blank panel. The panels with LDH and M-LDH-1 exhibit similar and minor improvements in terms of thermal-insulation properties, with T_eq_ values of 72 °C and 76 °C, respectively. In addition, the UV–NIR spectral reflectance of coating with M-LDH-2 is significantly higher than that of coatings with M-LDH-1 and LDH. The UV–NIR spectral reflectance agrees with the thermal-insulation experiments. It is evident from the experiment that MTS-modified LDH using one-step in situ hydrothermal method displays the largest particle size and particle size distribution and demonstrates the best thermal-insulation effects. Conversely, the LDHs modified using the two-step method have smaller particle sizes and average thermal-insulation effects. In summary, this paper presents innovative and eco-friendly LDH-based thermal-insulation coatings.

## Figures and Tables

**Table 1:** Formulation of the thermal insulating coatings.

Ingredients	W_t_ (%)
Acrylic resin	30
Film-forming additive	8
Fillers	48
Water	14
Curing agent	1

## Data Availability

Not applicable.
